# Polymorphisms in the 5′ upstream regulatory region of *p21*^*WAF1/CIP1*^ and susceptibility to oesophageal squamous cell carcinoma

**DOI:** 10.1038/srep22564

**Published:** 2016-03-02

**Authors:** Wenjun Yang, Yong Li, Tao Ning, Hong Cai, Zhiqiang Chen, Ying Dong, Yang Ke

**Affiliations:** 1 Key Laboratory of Carcinogenesis and Translational Research (Ministry of Education), Department of Genetics, Peking University School of Oncology, Beijing Cancer Hospital and Institute, Beijing 100142, P. R. China; 2 Key Laboratory of Fertility Preservation and Maintenance (Ministry of Education), Cancer Institute of the General Hospital, Ningxia Medical University, Yinchuan, Ningxia, 750004, P. R. China; 3 Department of Laboratory Animal, Peking University School of Oncology, Beijing Cancer Hospital and Institute, Beijing 100142, P. R. China; 4 Radiology Department of General Hospital, Ningxia Medical University, Yinchuan, Ningxia, 750004, P. R. China

## Abstract

This study aims to scan the 5′-upstream regulatory region of the *p21* gene to identify all putative functional single nucleotide polymorphisms (SNPs) and to evaluate the contribution of *p21* variants to oesophageal squamous cell carcinoma (ESCC) in the Chinese Han population. Common SNPs were identified, and both locus-based and haplotype-based association tests were used to evaluate the potential risk of these *p21* gene polymorphisms for ESCC. Immunohistochemistry assay was further performed to detect the P21 protein expression in ESCC specimens. Twenty three SNPs were identified and seven Tagging SNPs were chosen to represent all 23 SNPs. Univariate analysis indicated that the rs3829963 C and the rs2395655 G alleles increased susceptibility to ESCC (OR = 1.606 and OR = 1.572, respectively). The rs3829963 C and rs2395655 G alleles, combined with cigarette smoking, could further increase the risk for ESCC (OR = 2.657 and OR = 2.828, respectively). Additionally, the rs2395655 G allele appeared to elevate the positive rate of P21 expression in ESCC tissues, as compared to the A allele. This report demonstrates for the first time that rs3829963 and rs2395655, in the promoter of the *p21* gene are potentially functional, modulating susceptibility to ESCC among the high-risk cigarette-smoking Chinese population.

Oesophageal cancer is the sixth most common cause of cancer-related deaths worldwide^1^ and the fourth among both men and women in China^2^. The incidence of oesophageal cancer has been found to show marked geographical variation. Anyang County in Henan Province of China, located in the southern portion of the Taihang Mountains, exhibits the highest incidence and mortality rates of oesophageal squamous cell carcinoma (ESCC) globally^3^. Environmental and genetic factors have been implicated in oesophageal carcinogenesis^4^,^5^. In high-risk areas, ESCC tends to cluster strongly in families^6^,^7^, suggesting that genetic susceptibility contributes to the high rates of ESCC in these areas^8^,^9^,^10^, in combination with exposure to environmental risk factors, including smoking, alcohol drinking, and nutritional deficiencies, among others. Despite extensive research, the aetiology of ESCC remains inconclusive. Some of the most promising candidate genes appear to be involved in the regulation of cellular activity. The *p21*^*WAF1/CIP1*^ gene (OMIM: 116899; hereafter, *p21*), a main downstream effecter of p53, is one of these promising candidate genes that have been shown to play vital roles in carcinogenesis. Because more than 50% of human cancers exhibit p53 mutations^11^, it is conceivable that the existence of natural variants or mutations in the *p21* gene could also be linked to the development of specific cancers. The natural genetic variants of the *p21* gene have therefore emerged as a resource for studies aimed at understanding differences in cancer risk between individuals. By scanning the coding region of the *p21* gene, our group previously demonstrated that only three SNPs existed in the coding region and 3′ untranslated region (3′ UTR) in a Chinese population^12^. Many previous reports investigating the effects of *p21* polymorphisms on cancer susceptibility have also mainly concentrated on these three SNPs^13^,^14^. It has been reported that P21 expression can be controlled at the transcriptional level by both p53-dependent and p53-independent mechanisms. That is, apart from p53, a variety of other factors are known to activate or repress P21 expression by binding to specific *cis*-acting elements located within the *p21* promoter region^15^, including Sp1/Sp3, Smads, Ap2, and E2F. Therefore, sequence variations, such as single nucleotide polymorphisms (SNPs) in the *p21* promoter region, likely alter P21 expression and lead to abnormal biological responses, including cancer.

In the present study, we first scanned the 5′-upstream regulatory region of the *p21* gene to identify all putative functional polymorphisms within 96 Chinese Han people from Henan Anyang County. The pattern of linkage disequilibrium (LD) across the 5′-upstream regulatory region was characterised, and tagging SNPs (tSNPs) were selected. Then, these tSNPs were further genotyped in the overall study population. Both locus-based and haplotype-based association tests were employed to evaluate any potential association of the *p21* gene with ESCC.

## Results

### SNP identification

A total of 23 polymorphisms were detected in the 5′-upstream regulatory region of the *p21* gene. In the 5′ flanking region, 13 polymorphisms were identified upstream of the transcriptional start point (rs4135234–rs4135239). No polymorphism was discovered in the first exon. Ten polymorphisms were identified in intron 1 (rs3176320–rs3176337). Position information and mutation types for all SNPs are presented in table 1, along with the minor allele frequencies. No significant deviation from Hardy-Weinberg equilibrium was observed for any polymorphism.

### Linkage disequilibrium, haplotype structure and selection of tSNPs

**Figure 1:**
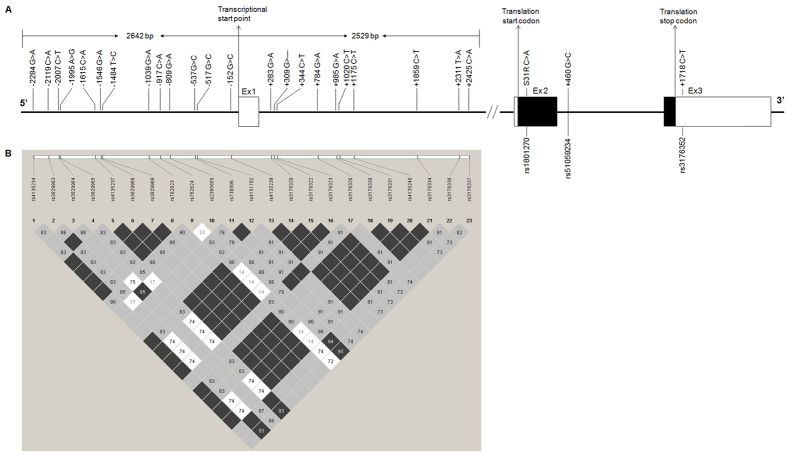
*p21* gene structure and linkage disequilibrium (LD) map for the SNPs in the promoter region of *p21*. genotyped in the current study. (**A**) Gene map and polymorphisms in the *p21* gene on chromosome 6p21.2. Coding exons are indicated with black blocks and 5′- and 3′-UTRs with white blocks. The first base of the transcription start site is denoted as  + 1. (**B**) LD blocks between SNPs in the promoter region of *p21* in 96 healthy Han Chinese individuals. Each diamond represents the correlation (D′) between each pair of SNPs. A standard colour scheme is used to display the LD pattern, where dark grey indicates very strong LD; white indicates no LD; and bright grey and shades of grey represent intermediate LD, with an increasing intensity of grey indicating an increasing degree of LD.

Figure 1 displays the pairwise LD coefficients (D′) of the 23 SNPs. Overall, the variants in this 5 Kb region exhibited strong LD. Because pairwise LD tests within a gene are not independent, the *P* values of the D′ values were adjusted based on the number of SNP pairs in the *p21* gene using an FDR correction. Notably, most of the polymorphisms in the 5′-upstream regulatory region (rs4135234–rs4135237–rs3829966–rs3829968–rs762623–rs4135239–rs3176330–rs3176331, rs3829963–rs3829965, rs730506–rs4151702–rs3176326, and rs3176320–rs3176322–rs3176323–rs4135240–rs3176334) were in perfect LD with one another.

Table 2 shows the inferred haplotypes (frequency >1%) of the 23 SNPs in the 96 subjects. Four truly common haplotypes each presented a frequency of over 10%, constituting 70% of the total. The frequencies of the remaining three haplotypes observed were approximately 5%.

After screening, a total of seven tSNPs (rs4135234, rs3829963, rs3829964, rs762624, rs2395655, rs730506, and rs3176320) were selected that showed a D′ >0.95 using Haploview software, which were the same as those that exhibited an R^2^H threshold of 0.9 using GOLD software (Table 2).

### Genotypes and risk of ESCC

Seven tSNPs were genotyped in all subjects. The characteristics of all subjects and the genotype distributions of the tSNPs of the *p21* gene are shown in Tables 3 and 4. No significant differences were found in age or gender (both *p* > 0.05) between the cases and controls. However, the distribution of cigarette smoking and alcohol consumption between the cases and controls was significantly different (both *p* < 0.05).

Single SNP analyses indicated that both rs3829963 and rs2395655 were significantly associated with ESCC. Compared with rs3829963 AA, the rs3829963 C allele was associated with higher susceptibility to ESCC, even after adjustment for multiple conventional ESCC risk factors (OR = 1.606, 95% CI = 1.148–2.247, *p*  < 0.05). Similarly, the rs2395655 G allele was associated with higher susceptibility to ESCC compared with rs2395655 AA homozygotes, even after adjustment for multiple conventional ESCC risk factors (OR = 1.572, 95% CI = 1.129–2.190, *p* < 0.05). No association was found with the other tSNPs (Table 4).

After eliminating rare haplotypes (with an estimated frequency of <0.03), 7 remaining haplotypes required evaluation. The haplotype-based test was performed using the haplo.stats program. For haplo.score analysis, *p* = 2×10^−5^ for the global distribution of all haplotypes, with 9 degrees of freedom (df) between cases and controls, which was identical to the empirical *P* value (*p* = 0.01) based on 1,000 simulation repetitions. Haplotype-based association analyses revealed that the frequency of Hap 1 GACCAGA was lower in cases than in controls (11.3% *vs*. 14.3%, *p* = 0.036) after adjusting for covariates (Supplemental Table 2).

To identify possible risk haplotypes and evaluate the effect of each haplotype, we ran the haplo.glam model, in which Hap 1 GACCAGA, the protective haplotype, was chosen as the baseline. The results of model fitting are shown in supplemental Table 3. Compared with this haplotype, no other haplotypes were found to significantly increase the risk of ESCC (*p* > 0.05).

We further statistically investigated the association analyses of the rs3829963 or rs2395655 genotypes and other potential risk factors, including smoking and alcohol consumption, on the risk of ESCC. Using non-smokers carrying rs3829963 AA as a reference, smokers harbouring the AA genotype (OR = 1.845, 95% CI = 0.953–3.576) and non-smokers with the CA or CC genotype (OR = 1.460, 95% CI = 1.002–2.174) were found to display an increased risk for ESCC. Furthermore, smokers with the CA or CC genotype presented a significantly increased risk for ESCC (OR = 2.657, 95% CI = 1.702–4.149). Similarly, using non-smokers carrying rs2395655 AA as a reference, smokers with the AA genotype (OR = 2.068, 95% CI = 1.109–4.248) and non-smokers with the GA or GG genotype (OR = 1.581, 95% CI = 1.059–2.360) were observed to show an increased risk for ESCC. Furthermore, the crossover analyses showed that smokers with the GA or GG genotype exhibited a significantly increased risk for ESCC (OR = 2.828, 95% CI = 1.818–4.389) (shown in Table 5). However, no significant result was observed between the tSNPs and alcohol consumption via logistic analysis (*P* > 0.05, data not shown).

### Association of the rs3829963 or rs2395655 genotypes with the P21 expression of ESCC patients

**Figure 2:**
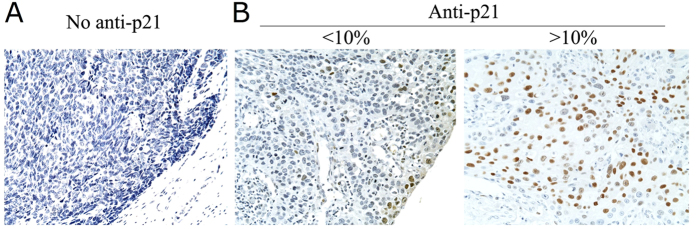
Immunohistochemistry staining of the P21 protein in ESCC tissues. (**A**) No–anti p21, negative control with primary antibody replaced by PBS. (**B**) Anti–p21, the brown signals represent positive staining for P21 protein and the staining was scored on a scale as indicated in the Materials and Methods. Positive cases were defined as those with over 10% of examined cells stained. Magnification, ×400.

To further analyze the effects of the rs3829963 or rs2395655 genotypes on P21 expression in human tissues, we examined the expression of P21 protein in 93 cases of ESCC tissues. The representative examples of P21 staining negative, positive I and above were shown in Fig. 2. The brownish signals represent the positive staining and were found mainly in the nucleus of tumor cells and the para-basal layer of normal esophageal mucosa. As shown in Table 6, 34 (36.6%) out of 93 ESCC specimens were scored P21 staining positive I and above. The positive rate of P21 protein was much higher in ESCC tissues carrying the rs2365955 G allele than tissues with the A allele (31.2% vs. 5.4%; *p* = 0.001), indicating significant association between the rs2365955 genotypes and P21 protein expression. However, no association was shown between the rs3829963 genotypes and P21 expression (*p* > 0.05).

## Discussion

The *p21* gene, located on chromosome 6p21.2, is also known as WAF1, CIP1, SDI-1, MDA6, and Cap20 because its cDNA was cloned independently by several groups using a number of different screening strategies^16^. The gene consists of three exons and two introns and encodes a 21 kDa protein. The translation region lies mainly in exon2^17^. As the first cyclin-dependent kinase inhibitor (CDKI) to be identified to date, *p21* can bind cyclin/CDK complexes and negatively modulates cell cycle progression. It is also part of quaternary complexes that contain cyclin, CDK, and PCNA, a processivity factor for DNA polymerase δ (Polδ). The formation of these complexes is essential for cell cycle progression. Through a direct interaction with PCNA, *p21* prevents DNA synthesis and regulates DNA methylation^18^. Varieties of transcriptional factors activate or repress P21 expression by binding to specific *cis*-acting elements located within the *p21* promoter region^16^. Naturally occurring SNPs in the *p21* promoter likely have a great impact on its transcriptional activity by altering its ability to bind to certain nuclear proteins. In the present study, we applied tSNP selection and haplotype-based analysis methods to identify functional SNPs in the 5′-upstream regulatory region of the *p21* gene in a Chinese population and investigated their association with the risk of developing ESCC. This was the first systematic scanning study of polymorphisms in the 5′-upstream regulatory region of the *p21* gene in a Chinese population.

Deletion analysis of the *p21* promoter revealed a distal (positions -2,300 to -2,100 bp) and a proximal (positions −124 to −61 bp) region that act synergistically to achieve high levels of constitutive expression^19^. Two p53 binding regions have also been previously confirmed at approximate positions of −1,400 bp and −2,300 bp^20^. Therefore, we screened the 5′ flanking region up to 2.5 Kb upstream and the region extending 2.6 Kb downstream from the transcriptional start site. We identified 23 polymorphisms within a distance of just over 5 Kb from the *p21* gene. We also found that complete LD or even perfect LD existed among most of these variants, indicating that all of polymorphisms might exist in one large haplotype block within the 5′-upstream regulatory region of the *p21* gene. Using tSNPs software, we chose seven tSNPs (rs4135234, rs3829963, rs3829964, rs762624, rs2395655, rs730506, and rs3176320) to represent all other SNPs and subsequently detected them within the overall study population. Analyses were conducted based on both single SNPs and haplotypes. The single SNP univariate analysis indicated that the rs3829963 C allele and the rs2395655 G allele conferred risk for ESCC, even after adjustment for multiple conventional ESCC risk factors, and they may therefore likely correspond to susceptible forms of ESCC. We also found that the rs3829963 C allele and the rs2395655 G allele, combined with cigarette smoking, further increased the risk for ESCC. Haplotypes are the particular combinations of alleles observed in a population. In this report, haplotype-based association analyses revealed that the frequency of the GACCAGA haplotype was lower in cases than in controls after adjusting for covariates, indicating that the GACCAGA haplotype may play a protective role in ESCC.

In the present study, we confirmed 23 SNPs in the 5′-upstream regulatory region of the *p21* gene in a Chinese population. In general, mutations in biologically important genes may readily lead individuals to a high risk for diseases under balanced selection. Therefore, embryonic cells carrying these mutations may die off in an early stage, and only those mutations with mild effects on biological functions will be maintained and transmitted to daughter cells, some of which may inevitably result in susceptibility to diseases under certain living conditions. By scanning most of the *p21* gene in a Chinese population, our group identified 3 polymorphisms within 3 Kb of the coding region and 23 polymorphisms within 5 Kb of the regulatory region, which suggested that the coding region of *p21* was highly conserved, while still presenting numerous variants in the promoter region. Although all 23 polymorphisms have been recorded in the NCBI SNP database, we report the first association with ESCC in a Chinese population to the best of our knowledge. In this study, we also found that cigarette smoking was a risk factor for ESCC. When smoking was combined with the rs3829963 C allele or the rs2395655 G allele, the risk for ESCC increased further. We used 93 cases of paraffin-embedded ESCC specimens and performed immunohistochemistry assay to detect the P21 protein expression. In these ESCC specimens, positive rate of the P21 protein expression in ESCC tissues with the rs2365955 G allele was raised obviously in comparison with the A allele, suggesting that the rs2365955 genotypes were associated with the P21 protein expression in ESCC tissues.

All in all, this was the first systematic polymorphism scanning study in *p21* gene in Chinese ESCC population, which provided us some valuable information about two potential functional SNPs. However, there are some limitations in our work. First, all SNPs have only been tested in ESCC population in limited samples. Therefore, replication in a larger sample of ESCC or other cancers is still required. Second, since all our subjects, including patients and controls were only selected from either the Henan Anyang Cancer Hospital or communities of Henan Province, China, selection bias cannot be ignored. Last, further functional work is also needed, which will provide additional information about the functional polymorphisms in cancers.

## Materials and Methods

The workflow chart of the whole study was showed in Supplemental Figure 1.

### Subjects for the case-control Study

A total of 490 patients with ESCC and 600 cancer-free controls were enrolled in this study. All of the subjects were unrelated ethnic Han Chinese. Patients with histopathologically confirmed ESCC were recruited from January 2004 to December 2007 at the Anyang Cancer Hospital of Henan Province, China. Demographic data, including the age at diagnosis, gender, cigarette consumption and alcohol use of the subjects, were obtained from medical records. Subjects with previous cancer and those who had received chemotherapy or radiotherapy were excluded.

Controls matching the cases for gender and age (± 5 years) were randomly selected in the same regions as the patients from a community-based cancer screening program for the early detection of oesophageal cancer. The recruited subjects were asymptomatic residents with ages from 40 to 75 years with no contraindications regarding oesophageal cytology or endoscopic examinations. Eligible subjects who agreed to participate and signed an informed consent form were assigned a study identification number and referred for examinations. Each subject was interviewed to obtain demographic and risk factor information and underwent an oesophageal balloon cytology examination and an endoscopy examination. Exfoliated oesophageal cells were collected in the balloon cytology examinations. Cells on the surface of the balloon were smeared onto slides for cytological diagnosis.

### Blood samples from all subjects were collected for DNA preparation and p21 gene genotyping

All experimental protocols in this study were approved by the Peking University Ethical Review Committee. All the methods were carried out in accordance with the approved guidelines All participants provided written informed consent according to local regulations.

### SNP identification

Searches for SNPs were performed in DNAs from 96 blood samples, which were randomly selected from 600 cancer-free subjects. These samples included 192 chromosomes, providing at least a 95% confidence level for the detection of alleles with frequencies >1%. A total of 5,171 bp were screened, including the 5′ flanking region up to 2,642 bp upstream of the transcriptional start site and extending 2,529 bp downstream of the transcriptional start site.

The 5′ flanking region starting 2,642 bp upstream of the transcriptional start site, the first exon, and the exon 1/intron 1 boundary were screened via direct DNA sequencing. Five sets of PCR primers were designed for SNP screening with Oligo 6.0, and five fragments were amplified from each subject (Supplemental Table 1). PCR was performed in a 25 μl reaction mixture containing 50 ng of DNA, 0.1 μM each primer, 0.2 mM dNTPs, 2.0 mM MgCl_2_, 0.5 units of Taq DNA polymerase (Promega, Madison, WI), and 1 × reaction buffer. The PCR profile consisted of an initial melting step of 2 min at 95 °C, followed by 35 cycles of 45 s at 94 °C, 40 s at 59–62.5 °C (variable Tm values according to different fragments), and 45 s at 72 °C, with a final elongation step of 10 min at 72 °C. SNPs were identified by directly sequencing the obtained PCR products with ABI PRISM Dye Terminator Sequencing kits (Applied Biosystems, Foster City, CA) and loading the samples onto an ABI 3700 sequencer. SNP candidates were then confirmed and analysed by two independent observers. We further confirmed these SNP positions and genotypes by re-amplifying and re-sequencing the SNP sites from the opposite strands.

We compared the SNPs which were detected in the direct DNA sequencing with those in the dbSNP-NCBI in the same region (2642 bp upstream, the 1st exon, boundary of exon 1 and intron 1). All detected SNPs were the same as SNPs in dbSNP. Therefore, to screen GC-rich intron1 region for polymorphisms, SNPs (with frequency >1%) were selected from the dbSNP-NCBI (http://www.ncbi.nlm.nih.gov/entrez/query.fcgi?db=snp) public database and further confirmed using Beckman GenomeLabTM SNPstream UHT (Ultra-High Throughput) Genotyping System (Beckman/Coulter, Fullerton, Calif.) by Shanghai Bio Star Genechip Inc.. Primers and probes were supplied directly by Beckman Coulter. Sequences are available on request. The target genomic sequences containing the SNPs of interest were amplified in 12-plex PCR reactions. After enzymatic clean-up, PCR products were subjected to an extension reaction using 5′-tagged extension primers and fluorescent dye-labeled terminators. In a thermal cycled extension step, the primers hybridized to the specific amplicons 1 base adjacent to the SNP site and were extended by one base at the 3′ end with a fluorescently labeled nucleotide. These reaction products were transferred to an array plate, where each of the 12 extension products in the multiplex reaction was sorted by hybridization of the unique 5′-tag sequence to its complementary probe immobilized in a mini-array within each well. The reader imaged the microarray plates. The 2-color detection allowed SNP detection by comparing signals from the 2-fluorescent dyes. The image signals were then transferred to genotyping software that translated the images of the arrays into genotype calls. The error rate on the basis of blind replicates was 0.1% to 2.0% for the SNPs examined in this study.

### Linkage disequilibrium, haplotype structure and selection of tSNPs

All patterns of pairwise LD among the SNPs and the correlation coefficient D′ for each pair of polymorphic sites were measured using both Haploview software (version 4.1, http://www.broad.mit.edu/mpg/haploview) and GOLD software (http://www.sph.umich.edu/csg/abecasis/GOLD/). Haplotype frequencies were estimated using the ‘partition-ligation’ E-M algorithm, as implemented in the tSNPs program of GOLD software. The tSNPs software was used to identify an optimal set of tSNPs that optimise the predictability of common haplotypes utilising the R^2^_H_ statistic. We ran the program with the following parameters: common haplotypes were defined as the minimal set of haplotypes that covers 70% of existing haplotypes, and sets of tSNPs resolving the common haplotypes were selected at an R^2^_H_ threshold of 0.9 21,22.

### Genotyping

Genomic DNA was obtained from peripheral blood lymphocytes from all controls and patients via standard phenol-chloroform extraction. Seven tSNPs were selected and genotyped from all subjects. All genotyping was performed via direct DNA sequencingor single-base-pair extension on the Beckman GenomeLabTM SNPstream UHT Genotyping (Beckman/Coulter, Fullerton, Calif) platform. To assess genotype concordance, rs4135234 and rs3176320 were double-genotyped using both methods. All results were 100% concordant.

### ESCC tissue samples

To investigate the association of P21 protein expression and genotypes of rs 3829963 or rs 2365955, 93 patients, who were retrieved from the surgical patients of 490 ESCC patients mentioned above from the Anyang Cancer Hospital of Henan Province, China. The enrolled patients involved 72 men and 21 women and aged 39 to 75 years. In all of these cases, the primary treatment was surgical and no patients had suffered from severe postoperative complications. All the specimens had been routinely formalin-fixed, paraffin-embedded, serially sectioned at 4 μm in thickness.

### Immunohistochemical analysis

P21 protein expression in ESCC tissues was examined using S-P immunohistochemical method. Briefly, after routine deparaffinization and hydration, slides were treated with 1% hydrogen dioxide and then heated in EDTA (pH 8.0) for antigen retrieval. Following 10% goat serum blocking, tissue sections were then incubated with Mouse Anti-Human P21^*WAF1*^ protein, (Biogenex, San Raman, CA) at 4 °C overnight. After rinsing, sections were subsequently incubated with goat anti-mouse biotin-conjugated IgG for 15 min and then with streptavidin-peroxidase conjugate for 15 min. The signal was developed with diaminobenzidine, and slides were counterstained with 5% hematoxylin. The brown signals located in the nuclear represent positive staining for P21 protein. Sections without monoclonal antibody treatment were used as negative controls. The staining was scored on a scale from 0 to III as follows: 0, less than 10% cells were stained; I, 10–24% cells were stained; II, 25–49% cells were stained; and III, 50–100% cells were stained. Scores I–III were classified as positive, while score 0 was negative.

### Association analyses

The main purpose of our analyses was to test the association of tSNPs or haplotype variation in the *p21* gene with ESCC. Analyses were conducted separately for each tSNP, followed by haplotype analyses.

Descriptive statistical analyses and unconditional logistic regression were performed with Statistical Analysis System software (version 8.01; SAS Institute, Cary, NC). Hardy-Weinberg equilibrium was tested by comparing the expected and observed genotype frequencies using χ^2^ statistics. Logistic regression analyses by dominant model were used to estimate the association between genotypes and ESCC risk. Odds ratios (ORs) were adjusted for gender, age, cigarette smoking, and alcohol drinking. Crossover analyses were used to conduct association analyses by cigarette smoking or alcohol drinking status. A *P* value of <0.05 was used as the criterion for statistical significance, and all statistical tests were two-sided. To test the association of statistically inferred haplotypes with ESCC, we used the haplo.score approach outlined by Schaid *et al*.^23^. This method assigns a probability to each haplotype pair in each individual and then directly models the phenotype of the individual as a function of each inferred haplotype pair, weighted by their estimated probability to account for haplotype ambiguity. This program can adjust for covariates and compute simulation *P* values both globally and for each haplotype. The number of simulations for empirical *P* values was set to 1,000. Because haplo.score does not provide the magnitude of the effect of each haplotype, haplo.glm was employed to calculate adjusted ORs and 95% confidence intervals (CIs) for each haplotype^24^. Both haplo.score and haplo.glm were implemented in haplo.stats software, developed using the *R* language^25^.

## Additional Information

**How to cite this article**: Yang, W. *et al*. Polymorphisms in the 5′ Upstream Regulatory Region of *p21^WAF1/CIP1^* and Susceptibility to Oesophageal Squamous Cell Carcinoma. *Sci. Rep*. **6**, 22564; doi: 10.1038/srep22564 (2016).

## Supplementary Material

Supplementary Information

## Figures and Tables

**Table 1:** Summary data on 23 polymorphisms discovered in 96 Chinese subjects.

Marker name (d bSNP name^a^)	Location in the chromosome	Region^b^	Position^c^	Alleles^d^	Minor alleles Frequency^e^(%)	Heterozygosity
SNP1 (rs4135234)	36752199	5′flanking	−2284	G/A	13.5	0.236
SNP2 (rs3829963)	36752364	5′flanking	−2119	C/A	49.0	0.50
SNP3 (rs3829964)	36752475	5′flanking	−2007	C/T	27.0	0.394
SNP4 (rs3829965)	36752488	5′flanking	−1995	A/G	49.0	0.50
SNP5 (rs4135237)	36752868	5′flanking	−1615	G/T	13.5	0.236
SNP6 (rs3829966)	36752929	5′flanking	−1546	C/T	13.5	0.236
SNP7 (rs3829968)	36752943	5′flanking	−1484	A/G	13.5	0.236
SNP8 (rs762623)	36753444	5′flanking	−1039	G/A	13.5	0.236
SNP9 (rs762624)	36753566	5′flanking	−917	C/A	38.5	0.474
SNP10 (rs2395655)	36753674	5′flanking	−809	G/A	47.0	0.498
SNP11 (rs730506)	36753946	5′flanking	−537	G/C	8.3	0.152
SNP12 (rs4151702)	36753966	5′flanking	−517	G/C	8.3	0.152
SNP13 (rs4135239)	36754331	5′flanking	−152	G/C	13.5	0.234
SNP14 (rs3176320)	36754766	5′UTR	283	A/G	22.0	0.343
SNP15 (rs3176322)	36754792	5′UTR	309	G/−	22.0	0.343
SNP16 (rs3176323)	36754827	5′UTR	344	T/C	22.0	0.343
SNP17 (rs3176326)	36755267	5′UTR	784	G/A	8.3	0.152
SNP18 (rs3176330)	36755468	5′UTR	985	G/A	13.5	0.234
SNP19 (rs3176331)	36755503	5′UTR	1020	C/T	13.5	0.234
SNP20 (rs4135240)	36755658	5′UTR	1175	C/T	22.0	0.343
SNP21 (rs3176334)	36756342	5′UTR	1859	C/T	22.0	0.343
SNP22 (rs3176336)	36756794	5′UTR	2311	T/A	27.0	0.394
SNP23 (rs3176337)	36756898	5′UTR	2415	C/A	22.0	0.343

^a^rs No. was shown if present in the dbSNP database.

^b^5′ flanking means the upstream from the first transcribed nucleotide.

^c^The base immediately preceding the start of transcription numbered as ‘−1’.

^d^With major allele given first and minor allele given second.

^e^Using 96 Chinese Han subjects.

**Table 2:** Haplotype structure of the *p21* gene in Chinese population.

HapID^a^	Haplotype	Frequency^b^	Cum freq^c^	R^2^_H_
A	G^*^A^*^C^*^GCGTGC^*^G^*^G^*^GGA^*^GTGGCCCTC	0.22	0.22	0.92
B	G^*^C^*^T^*^ACGTGA^*^A^*^G^*^GGA^*^GTGGCCCAA	0.19	0.41	0.97
C	G^*^A^*^C^*^GCGTGC^*^A^*^G^*^GGA^*^GTGGCCCTC	0.17	0.58	0.97
D	A^*^C^*^C^*^AAACAA^*^G^*^G^*^GCG^*^–CGATTTTC	0.12	0.70	0.95
E	G^*^C^*^C^*^ACGTGC^*^G^*^C^*^CGG^*^—CAGCTTTC	0.08	0.78	0.98
F	G^*^A^*^C^*^GCGTGC^*^G^*^G^*^GGA^*^GTGGCCCTC	0.05	0.83	–
G	G^*^C^*^C^*^ACGTGC^*^G^*^G^*^GGA^*^GTGGCCCTC	0.03	0.86	–

*Tagging SNPs.

^a^Haplotype designation.

^b^Frequency of the common haplotypes estimated by E-M algorithm.

^c^Cumulative frequency.

**Table 3:** Comparison of characteristics between cases and controls.

	Case (n = 490)	Control (n = 600)	*p*Value*
Mean age (yr±SD)	54.21±10.518	54.83±9.462	0.302
Gender (male/female)	270/220	306/294	0.177
Smoking (yes,%)	198 (40.4)	161 (26.83)	<0.001
Alcohol drinking (yes,%)	120 (24.49)	79 (13.17)	<0.001

Means±SD values for continuous variables.

^*^Two-sided χ^2^ test.

**Table 4:** Adjusted OR for the association of tSNPs with ESCC.

	Case	Control	OR 95%CI*	*p*value*
	(n = 490)	(n = 600)		
SNP1 (rs4135234)				
GG	385	481	1.000	–
GA	102	115		
AA	3	4		
GA+AA	105	119	1.083(0.794–1.478)	0.613
SNP2 (rs3829963)				
AA	70	124	1.000	–
CA	254	288		
CC	165	185		
CA+CC	419	473	**1.606(1.148–2.247)**	**0.006**
SNP3 (rs3829964)				
CC	179	245	1.000	–
CT	282	303		
TT	29	50		
CT+TT	311	353	1.190(0.919–1.541)	0.186
SNP9 (rs762624)				
CC	175	244	1.000	–
CA	253	268		
AA	62	86		
CA+AA	315	354	1.247(0.962–1.617)	0.095
SNP10 (rs2395655)				
AA	75	126	1.000	–
GA	294	295		
GG	120	179		
GA+GG	414	474	**1.572(1.129–2.190)**	**0.007**
SNP11 (rs730506)				
GG	380	460	1.000	–
GC	96	123		
CC	9	7		
GC+CC	105	130	1.007(0.742–1.368)	0.962
SNP14 (rs3176320)				
AA	259	335	1.000	–
AG	176	182		
GG	19	17		
AG+GG	195	199	1.265(0.967–1.656)	0.087

^*^Adjusted for age, gender, smoking and alcohol drinking status.

**Table 5:** Risk of ESCC associated with *p21* rs3829963 and rs2395655 by smoking status.

Smoking^a^	Genotype	*p21* rs3829963		OR(95%CI)	Genotype	*p21* rs2395655		OR(95%CI)
		ESCC% (N = 490)	Control% (N = 600)			ESCC% (N = 490)	Control% (N = 600)	
No	AA	8.8	16.2	Ref (1.000)	AA	8.6	15.0	Ref (1.000)
Yes	AA	5.7	5.3	1.845 (0.953–3.576)	AA	6.9	5.0	2.068 (1.109–4.248)
No	CA+CC	47.3	56.7	1.460 (1.002–2.174)	GA+GG	47.3	57.3	1.581 (1.059–2.360)
Yes	CA+CC	38.2	22.2	2.657 (1.702–4.149)^Δ^	GA+GG	37.1	21.7	2.828 (1.818–4.389)^Δ^

^Δ^*P*<0.01.

^a^Adjusted for age, gender, and alcohol drinking status.

**Table 6:** Association of rs3829963 and rs2365955 with P21 expression of ESCC patients.

P21 expresstion	rs3829963 no. (%)		p^a^	rs2365955 no. (%)		p^a^
	CC	CA+AA		AA	AG+GG	
negative	25 (26.9)	34 (36.5)	0.502	30 (32.2)	29 (31.2)	0.001
positive	12 (12.9)	22 (23.7)		5 (5.4)	29 (31.2)	

^a^Two-sided χ^2^ test.
