# Supplementary material for: Polymorphisms in the 5′ upstream regulatory region of p21WAF1/CIP1 and susceptibility to oesophageal squamous cell carcinoma
Source: Sci Rep. 2016 Mar 2;6:22564. doi: 10.1038/srep22564 (PMC4773838; doi:10.1038/srep22564)
Supplement: Supplementary Information [file srep22564-s1.pdf]

# Polymorphisms in the 5' Upstream Regulatory Region of *p21*$^{WAF1/CIP1}$ and Susceptibility to Oesophageal Squamous Cell Carcinoma

**Wenjun Yang [1,2], Yong Li[1,3], Tao Ning[1], Hong Cai[1], Zhiqiang Chen[4], Ying Dong[2], Yang Ke[1,*]**

[1]Key Laboratory of Carcinogenesis and Translational Research (Ministry of Education), Department of Genetics, Peking University School of Oncology, Beijing Cancer Hospital and Institute, Beijing 100142, P. R. China

[2]Key Laboratory of Fertility Preservation and Maintenance (Ministry of Education), Cancer Institute of the General Hospital, Ningxia Medical University, Yinchuan, Ningxia, 750004, P. R. China

[3]Department of Laboratory Animal, Peking University School of Oncology, Beijing Cancer Hospital and Institute, Beijing 100142, P. R. China

[4]Radiology Department of General Hospital, Ningxia Medical University, Yinchuan, Ningxia, 750004, P. R. China

[*]To whom correspondence should be addressed: Yang Ke, Key Laboratory of Carcinogenesis and Translational Research (Ministry of Education), Genetic Department, Peking University Cancer Hospital and Institute, Beijing 100042, P. R. China. Tel: 86-10-88196762; Fax: 86-10-88196735; E-mail: keyang@bjmu.edu.cn

**Figure legend: Supplemental Figure 1: Workflow chart of the study.**

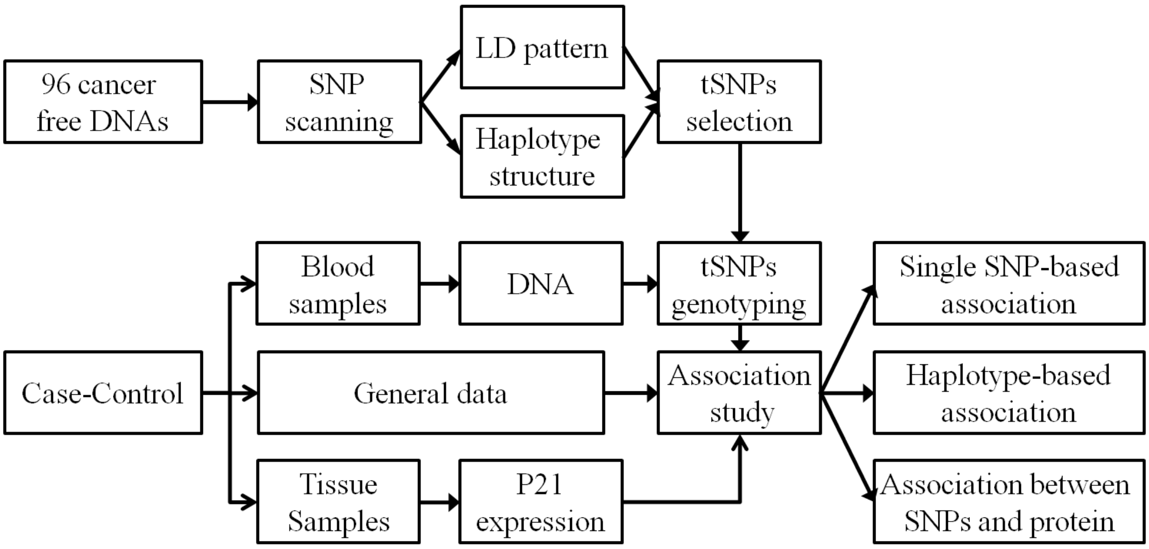


**Supplemental Figure 1: Workflow chart of the study.**

**Supplemental Table1: Primer sequences and Tm for amplifying the *p21* gene.**

| Fragment | Range | Primers (5'-3') | Length (bp) | Tm (℃) |
|---|---|---|---|---|
| 1 | 5'flanking | F: CTCTTCTCTGGGGTCTCACT<br>R: TCTTCTATGCCAGAGCTC | 468 | 56 |
| 2 | 5'flanking | F: TGTGGCTCTGATTGGCTTTC<br>R: GGGAGGACAGGCTTCTTTC | 892 | 61 |
| 3 | 5'flanking | F: GGCTTAGAGTGGGGTCCTG<br>R: GGCTCTCTGCTTGTCATCCT | 576 | 62.5 |
| 4 | 5'flanking | F: GCACAGAAAGGAGGCAAAG<br>R: AGGCAAGGATTTACCCAATG | 567 | 60.5 |
| 5 | 5'flanking+ exon1 | F: CATCTGTGAAATAAACGGGAC<br>R: GGAGCCAACCGCAGCCAAAG | 1069 | 65 |

**Supplemental Table 2: Distribution of *p21* gene haplotypes between cases and controls.**

| Haplotype[a] | | Frequency | | | Score | *p* value[b] | |
|---|---|---|---|---|---|---|---|
| | | ALL | Case | Control | | $\chi^2$ test | Simulation |
| Hap1 | G-A-C-C-A-G-A | 0.130 | 0.113 | 0.143 | -2.080 | 0.037 | 0.036 |
| Hap2 | A-C-C-A-G-G-G | 0.078 | 0.078 | 0.085 | -1.233 | 0.217 | 0.219 |
| Hap3 | G-A-C-C-G-G-A | 0.237 | 0.237 | 0.239 | -0.639 | 0.522 | 0.521 |
| Hap4 | G-C-C-C-G-C-G | 0.089 | 0.086 | 0.093 | -0.310 | 0.756 | 0.759 |
| Hap5 | G-C-T-A-A-G-A | 0.235 | 0.234 | 0.237 | -0.239 | 0.810 | 0.811 |
| Hap6 | G-C-T-C-A-G-A | 0.042 | 0.039 | 0.044 | 0.090 | 0.928 | 0.927 |
| Hap7 | G-C-C-C-G-G-A | 0.042 | 0.043 | 0.041 | 0.233 | 0.816 | 0.819 |
| Global Score Test[c] | | | | | | $2 \times 10^{-5}$ | 0.000 |

[a] Loci are arranged in the order rs4135234-3829963-3829964-762624-2395655-730506-3176320.

[b] Environmental covariates were adjusted.

[c] The global score statistic was tested with 9 df, adjusted for environmental covariates.

**Supplemental Table 3: Association between *p21* gene haplotypes and ESCC.**

| Model | Variable | Haplotype | Frequency | | Coefficient | *p* value |
|---|---|---|---|---|---|---|
| | | | Case | Control | | |
| Haplotypes* | Hap1 | GACCAGA | 0.113 | 0.143 | - | - |
| | Hap2 | ACCAGGG | 0.078 | 0.085 | 0.0984 | 0.665 |
| | Hap3 | GACCGGA | 0.237 | 0.239 | 0.27895 | 0.113 |
| | Hap4 | GCCCGCG | 0.086 | 0.093 | 0.28615 | 0.102 |
| | Hap5 | GCTAAGA | 0.234 | 0.237 | 0.30453 | 0.276 |
| | Hap6 | GCTCAGA | 0.039 | 0.044 | 0.22983 | 0.284 |
| | Hap7 | GCCCGGA | 0.043 | 0.041 | 0.2736 | 0.339 |

*Haplotype GACCAGA (Hap1) was chosen to be the baseline haplotype.
